# The chain mediation effect of rumination and worry between the intentionality and content dimensions of mind wandering and internalizing symptoms of depression and anxiety

**DOI:** 10.1038/s41598-025-99249-5

**Published:** 2025-07-01

**Authors:** Siqing Guan, Toru Takahashi, Nozomi Tomita, Hiroaki Kumano

**Affiliations:** 1 Research Area of Clinical Psychology, Graduate School of Human Sciences, Waseda University, Tokyo, Japan; 2 Japan Society for the Promotion of Science, Tokyo, Japan; 3 Laureate Institute for Brain Research, Tulsa, OK USA; 4 Faculty of Humanities and Social Sciences, Jissen Women’s University, Tokyo, Japan; 5 Comprehensive Research Organization, Waseda University, Tokyo, Japan; 6 Faculty of Human Sciences, Waseda University, Tokyo, Japan

**Keywords:** Mind wandering, Intentionality, Content, Rumination, Worry, Internalising symptoms, Psychology, Human behaviour

## Abstract

Rumination and worry, as maladaptive emotion regulation strategies, perpetuate internalizing symptoms of depression and anxiety. However, examinations of dysfunctional mind wandering (MW)—internally oriented thoughts that contribute to emotion dysregulation—and its underlying the cognitive mechanisms related to internalizing symptoms remains limited. This study aimed to identify dysfunctional types of MW associated with internalizing symptoms of depression and anxiety by distinguishing between the intentionality and content of MW. The results indicate that trait rumination and worry sequentially mediate the relationship between the frequency of unintentional MW, which encompasses vague, future-oriented, and negative content, and internalizing symptoms. Furthermore, both single and chained mediation effects were identified in the relationship between unintentional negative MW and internalizing symptoms, with worry acting either as a sole mediator or as a subsequent mediator following rumination in a chain mediation pathway. This study offers novel insight into the distinct roles of rumination and worry as maladaptive emotion regulation strategies, particularly regarding dysfunctional MW types and their contributions to internalizing symptoms of depression and anxiety.

## Introduction

Mind wandering (MW) refers to the phenomenon where attention shifts away from the current task or environment toward task unrelated thoughts^1^. MW is a default mental activity occurring daily and accounts for approximately 10ー50% of waking time^2–6^. While MW serves adaptive functions such as future planning, goal progress, and creative problem-solving^7–12^, it is also increasingly linked to negative affect, depression, and anxiety^4,13–15^. Internalizing symptoms, with 85–90% comorbidity between depression and anxiety, contribute to increased negative affect and emotion dysregulation^16,17^. However, the mechanisms linking dysfunctional forms of MW to internalizing symptoms of depression and anxiety remain poorly understood^13,14^.

### The content and intentionality of mind wandering

The content and intentionality of MW may play important roles in varying associations with internalizing symptoms of depression and anxiety. The content regulation hypothesis emphasizes the importance of MW content, such as temporal orientation, emotional valence, and vagueness, suggesting that MW can either disrupt or enhance mental health depending on its content^18^. Specifically, past- or future-oriented MW, as well as negative MW, are positively correlated with depressive and anxiety symptoms^19^. Additionally, distinguishing between intentional and unintentional MW is vital^20,21^. Intentional MW refers to a deliberate shift of attention from external tasks to internal thoughts, driven by higher-order (top-down) control or motivation^21,22^. In contrast, unintentional MW refers to an involuntary shift of attention, governed by lower-order (bottom-up) processes not influenced by motivation^21,22^. Intentional MW comprises future-oriented and positive content, whereas unintentional MW comprises past-oriented and negative content^23^. Unintentional MW is positively associated with depression, anxiety, and stress, whereas intentional MW is weakly negatively associated with anxiety and stress and is unrelated to depression^24^. The content and intentionality of MW critically influence its association with depression and anxiety, yet the specific mechanisms underlying this influence remain unclear.

### The mediating roles of rumination and worry

MW, categorized according to its intentionality and content, may contribute to internalizing symptoms of depression and anxiety through maladaptive emotion regulation strategies of rumination and worry. Research findings on the relationship between MW and rumination are inconsistent. Patients with Major Depressive Disorder (MDD) exhibit higher levels of rumination or worry and more frequent negative MW compared to healthy controls^25^. In contrast, studies involving patients with MDD and healthy controls have found that rumination is negatively correlated with MW, while rumination levels are closely linked to functional connectivity within neural networks associated with depression^26^. Additionally, although unintentional MW has been linked to rumination^27^, the temporal orientation of MW appears unrelated to rumination^28^. Therefore, investigating the role of rumination in the relationship between depression and combined dimensions of MW, particularly with regards to intentionality and content is essential. Regarding worry, literature reviews of MW and anxiety suggest that the relationship between MW and worry remains underexplored, despite worry being a key factor in anxiety^14^. Previous research has suggested that intentional MW is more likely to involve future-oriented or less vague content compared to unintentional MW^29^. However, the theoretical possibility that future-oriented intentional MWs are associated with planning, while future-oriented unintentional MWs are linked to worry, has been proposed and warrants further investigation^29^.As clarified previously, dysfunctional MW can be identified as unintentional MW, unintentional future-oriented MW, unintentional vague MW, and unintentional negative MW. However, whether rumination and worry play a role in maladaptive emotion regulation strategies in dysfunctional MW and internalizing symptoms is unclear.

### Conceptual distinction of mind wandering, rumination, and worry

 To examine emotion dysregulation between dysfunctional MW and internalizing symptoms, MW, rumination, and worry must be treated as distinct constructs^13,14^. Most theoretical studies have addressed MW, rumination, and worry as separate constructs^2,30^. However, previous research has often treated state-level rumination and worry as forms of state-level MW, failing to clearly distinguish between these constructs^13,14^. This lack of differentiation has contributed to conflicting findings regarding the relationships among MW, rumination, and worry^25,26,29^. As discussed above, if separating MW tendencies from rumination and worry at the state level is challenging, their differences at the trait level should be emphasized. The frequency of MW, assessed through multi-category thought sampling during experimental tasks and examined by item response theory latent modeling, provides insights into an individual’s MW tendencies across a wider range of the trait continuum^31^. Furthermore, by adding evidence of ecological validity, prior research has demonstrated that MW frequency observed during laboratory tasks can predict MW frequency in daily life even one year later, suggesting that MW tendencies represent a stable individual trait^32^. Moreover, rumination and worry have been conceptualized as dispositional maladaptive emotion regulation strategies ^33,34^. Gross’s (1998) process model identifies rumination and worry as antecedent-focused and response-focused emotion dysregulation strategies^35^. Rumination involves excessive fixation on internal events as part of a reappraisal process aimed at preventing negative thoughts or emotions^36–38^. In contrast, worry is characterized by cognitive avoidance that seeks to reduce rapid emotional reactions to distressing content^39^; it has been identified as a stronger predictor of anxiety and depressive symptoms than rumination^40^. Based on these conceptualizations, dysfunctional MW, rumination, and worry are assessed as trait-level distinct constructs, each contributing uniquely to the understanding of internalizing symptoms; however, their interrelationships at the trait level remain unexplored.

### The purpose of the current study

The present study focused on dysfunctional MW and internalizing symptoms of depression and anxiety, investigating the role of emotion dysregulation of rumination and worry. By clarifying the distinct contributions of MW, rumination, and worry, this study aimed to deepen the understanding of their roles in the maintenance and development of internalizing symptoms, providing a foundation for more targeted interventions.

Although prior research^24^ suggests weak or negligible negative associations between intentional MW and internalizing symptoms, unintentional MW has been more strongly and positively linked to these symptoms. The present study specifically targeted unintentional MW as a dysfunctional process, given its alignment with automatic cognitive mechanisms closely associated with executive control failures and subsequent emotion dysregulation. As a form of dysfunctional MW, unintentional MW is characterized by its automaticity and association with executive control failures, often involving future-oriented, vague, or negative content^23,29^. These deficits in executive control compromise attentional resources, thereby increasing vulnerability to maladaptive emotion regulation strategies of rumination and worry. Considering these emotional dysregulation processes, the high frequency of dysfunctional MW may contribute to the maintenance and development of internalizing symptoms by amplifying trait rumination and increasing trait worry. Specifically, dysfunctional MW may foster excessive fixation on internal events, thereby intensifying rumination, and may promote worry as a cognitive avoidance strategy in response to the distress it generates.

Specific hypotheses were as follows:

#### Hypothesis 1

The frequency of unintentional MW is positively and indirectly related to internalising symptoms serially mediated by rumination and worry.

#### Hypothesis 2

The frequency of unintentional future-oriented MW is positively and indirectly related to internalising symptoms serially mediated by rumination and worry.

#### Hypothesis 3

The frequency of unintentional vague MW is positively and indirectly related to internalising symptoms serially mediated by rumination and worry.

#### Hypothesis 4

The frequency of unintentional negative MW is positively and indirectly related to internalising symptoms serially mediated by rumination and worry.

## Materials and methods

### Participants and procedure

We analyzed data from 55 participants (20 males and 35 females; mean and standard deviation of age: 20.92± 2.23). The inclusion criteria were as follows: undergraduate and graduate students in good physical and mental health, as confirmed through a health questionnaire. The exclusion criteria were as follows: participants with a history of mental disorders, those who had not taken any medication within the past 24 h, those who had consumed alcohol within the past 12 h, or those who had consumed caffeine within the past 6 h prior to the experiment. Sixty undergraduate and graduate students from a university in Japan participated. They received payment of 2,500 Japanese yen each. Data from five participants were removed from all subsequent analyses because of operating errors during task execution. The sample size was based on a series of calculations conducted using G*Power 6. For the following sample size calculations, we used an alpha level of 0.05 to account for linear multiple regression analysis and a power level of 95%. To ensure that three predictor variables were present to explain 40–50% of the variance in internalising symptom scores (f^2^ = 0.35), 54 participants were required. Furthermore, considering the loss of data owing to interruptions in participation in the experiment and errors in execution, the experiment was designed for 60 participants. All experimental protocols were approved by the Ethics Committee for Research on Human Subjects of Waseda University in Japan (Approval No. 2019 − 131). All participants provided written informed consent. All methods were implemented in accordance with relevant guidelines and regulations.

### Questionnaires

#### Depressive symptoms

The Japanese version of the Center for Epidemiologic Studies Depression Scale(CES-D)was used to measure depression^41^. The CES-D is a 20-item self-report scale that measures the frequency of symptoms that have occurred during the previous week, with total scores ranging from 0 to 60. The internal consistency of the CES-D in this study was good (Cronbach’s α = 0.89). The mean and standard deviation of the CES-D scores were 17.27 and 9.78, respectively.

#### Anxiety symptoms

The Japanese version of the Generalised Anxiety Disorder-7 (GAD-7) was used to measure anxiety that occurred during the previous week^42^. Responses were scored on a four-point scale ranging from 0 (not at all) to 3 (nearly every day), with total scores ranging from 0 to 21 points. The internal consistency of the GAD-7 in this study was good (Cronbach’s α = 0.84). The mean and standard deviation of the GAD-7 scores in this study were 4.56 and 3.83, respectively.

#### Trait worry

The Japanese version of the Penn State Worry Questionnaire (PSWQ) was used to measure the level of trait worry^43^. The PSWQ is a 16-item self-report questionnaire that measures individual differences in the intensity and excessiveness of worry, with total scores ranging from 16 to 80. The internal consistency of the PSWQ in this study was excellent (Cronbach’s α = 0.94). The mean and standard deviation of the PSWQ scores were 54.00 and 13.96, respectively.

#### Trait rumination

The Japanese version of the Rumination-Reflection Questionnaire (RRQ) was used to measure dispositions towards rumination and reflection^44^. The subscale RRQ- Rumination was suitable for the current study to measure trait rumination. The internal consistency of the RRQ- Rumination in this study was excellent (Cronbach’s α = 0.92). The mean and standard deviation of RRQ- Rumination in this study were 41.62 and 10.03, respectively.

### Sequential sustained attention to response task (sequential SART)

Sequential SART is a cognitive task commonly used to measure MW by assessing lapses in sustained attention and inhibitory control during sequential stimulus processing^20,45^. The Sequential SART, with its lower cognitive load compared to the standard SART, is more likely to induce both intentional and unintentional MW^20,45^. We used the Sequential SART^20,45^with thought sampling to reduce memory biases inherent in self-reporting and assess individual differences in MW traits, categorized by their intentionality and content. Sequential SART was programmed using PsychoPy2, adapted from Seli et al. (2016) and Arabaci & Parris (2018)^20,45^. During each trial, digits 1‒9 were first presented in white in the centre of the black screen for 250 ms, and then an ‘x’ mark was presented for 900 ms (total trial duration = 1150 ms). In each block, the digits were presented in sequential order (1–9, repeated in each block), five possible font sizes (120, 100, 94, 72, and 48 points) were presented twice, and one appeared once (determined randomly). The digits were set using the Courier New typeface. Participants were required to press a space key for each digit, but were asked to withhold responses to digit 3. After 18 practice trials (two blocks), participants completed 900 trials (100 blocks).

### Thought sampling of dysfunctional MW

Multi-category thought sampling of the dysfunctional MW Trait was used to assess the frequency of intentionality and content using thought probes during sequential SART. The frequency of MW, assessed through multi-category thought sampling and analyzed using item response theory latent modeling, reflects an individual’s MW tendencies, with confirmed reliability and validity^31^. To ensure that the thought probe was provided after several trials, the experimental trials consisted of 18 trials (two blocks) with no thought probe. The thought probe was randomly presented 20 times between the 2nd to 98th blocks. Additionally, if a probe was presented during the same block, no more probes would be presented in the same block. When a probe occurred, the task stopped, and the participant was presented with the following question in Japanese: ‘Q1: Which of the following responses best characterises your mental state just prior to the presentation of this screen?’ The possible response options were ‘(1) On task’, ‘(2) Intentional MW’, and ‘(3) Unintentional MW’. Participants were instructed to respond via keypress (1–3). After providing a response to the thought probe, participants were presented with the following question in Japanese: ‘Q2: Which of the following responses best characterises your mental state just prior to the presentation of this screen, in terms of whether the content was related to the past or to the future?’ The possible response options were ‘(1) My thoughts involved future events’, and ‘(2) My thoughts involved past events’. Participants were instructed to respond via key press for future or past-oriented content (1–2), or to press the space key for completing the task.

After providing a response to Q2, participants were presented with the following question in Japanese ‘Q3: Which of the following responses best characterises your mental state just prior to the presentation of this screen, in terms of whether the content was positive, neutral, or negative?’ The possible response options were ‘(1) The content of my thoughts was positive’, ‘(2) The content of my thoughts was neutral’, and ‘(3) The content of my thoughts was negative’. Participants were instructed to respond via key press (1–3) or press the space key to complete the task. After providing a response to Q3, participants were presented with the following question in Japanese ‘Q4: Which of the following responses best characterises your mental state just prior to the presentation of this screen, in terms of whether the content was specific, or vague?’ The possible response options were ‘(1) The content of my thoughts was specific’, and ‘(2) The content of my thoughts was vague’. Participants were instructed to respond via key press (1–2) or to press the space key for being on task. Following 18 practice trials, participants completed 900 experimental trials with 20 thought probes. To eliminate the potential for response bias, these questions were presented to participants regardless of whether they responded that they were ‘on task’ in Q1. According to Seli et al. (2016) and Arabaci & Parris (2018), if participants were not required to press a response button for the questions (Q2 and Q3) after responding with an ‘on task’ response, they might have had a response bias to select the ‘on task’ response for minimal effort to accelerate the task, even if they were not actually on task^20,45^.

### Procedure

After providing informed consent and completing a set of computerised questionnaires, all participants performed the SART after measuring working memory capacity and executive function via four tasks for other research purposes. Before starting the SART, participants were also given detailed instructions on thought-probe responses (instructions were prepared in Japanese with reference to Seli et al. (2016)^20^. Before running the SART, the following definitions were given to participants: ‘on task’ meant that they were either not thinking or thinking about something related to the task (e.g., thinking about their performance, numbers, or their reactions), and ‘MW’ meant that they were thinking about something completely unrelated to the task (e.g., thinking about what to have for dinner, plans with friends, an upcoming test, etc.). Furthermore, participants were asked to indicate whether the MW was intentional (deliberate) or unintentional (spontaneous). After providing a response to Q1, participants were presented with two questions about their thought content just before the thought-probe presentation. Task performance included the percentage of accurate responses to target trials and the response time (in ms) for target trials. Additionally, the frequency of the MW was measured for each participant by counting the number of thought probes to which the participants reported each type of MW.

### Statistical analysis

Regarding internalising symptoms, each type of MW was used as an independent variable, rumination and worry were used as mediating variables, and the principal component scores of internalising symptoms consisting of depression and anxiety were used as the dependent variables. Principal component analysis extracted one component (eigenvalue: 1.76) that explained 87.96% of the total variance. The factor loadings of the two items on this component indicated that depression (factor loading = 0.94) and anxiety (factor loading = 0.94) loaded significantly onto one component. IBM SPSS Statistics 29.0. SPSS PROCESS macro version 4.3^46,47^ was used to test the mediation model. We computed 95% confidence intervals for the indirect effects obtained from the bias-corrected bootstrap estimates with a sample size of 5,000. These intervals are deemed significant at a p-value of 0.05 if the range of the 95% confidence interval does not encompass zero.

## Results

Table 1 presents the descriptive statistics of each variable and the correlations of intentional and unintentional MW with content, internalizing symptoms, rumination, and worry.

**Table 1:** The descriptive statistics of each variable and the correlations of intentional and unintentional MW with content, internalizing symptoms, rumination, and worry (*N* = 55).

MW type (M ± SD)		Internalising symptoms	Rumination	Worry
Intentional MW	(2.87 ± 3.43)	− 0.206	− 0.039	− 0.270*
Intentional past-oriented MW	(0.93 ± 1.09)	− 0.011	− 0.026	− 0.343*
Intentional future-oriented MW	(1.95 ± 3.02)	− 0.230	− 0.034	− 0.184
Intentional positive MW	(0.76 ± 1.29)	− 0.162	− 0.166	− 0.359**
Intentional neutral MW	(1.33 ± 1.86)	− 0.198	− 0.149	− 0.279*
Intentional negative MW	(0.78 ± 2.01)	− 0.065	0.179	0.026
Intentional specific MW	(1.75 ± 2.34)	− 0.232	− 0.137	− 0.293*
Intentional vague MW	(1.13 ± 2.27)	− 0.073	0.083	− 0.108
Unintentional MW	(7.95 ± 4.78)	0.301*	0.345**	0.197
Unintentional past-oriented MW	(3.24 ± 3.20)	0.099	0.161	− 0.114
Unintentional future-oriented MW	(4.71 ± 3.59)	0.313*	0.316*	0.364**
Unintentional positive MW	(1.98 ± 2.71)	0.102	0.031	0.029
Unintentional neutral MW	(4.42 ± 3.45)	0.135	0.240	0.001
Unintentional negative MW	(1.55 ± 1.98)	0.352**	0.372**	0.434**
Unintentional specific MW	(3.06 ± 2.71)	0.156	0.120	0.026
Unintentional vague MW	(4.89 ± 3.47)	0.293*	0.381**	0.251

Note: *p < 0.05, ** *p* < 0.01; The internalizing symptoms were calculated as a composite score of depression and anxiety scale scores. Descriptive statistics are as follows: Depression (M = 17.27, SD = 9.78); Anxiety (M = 4.56, SD = 3.80); Rumination (M = 41.62, SD = 10.03); Worry (M = 54.00, SD = 13.96).

### Chain mediation model of rumination and worry on the relationship between unintentional MW and internalising symptoms

By testing the mediation models, we first computed the model for rumination as the first mediator (M1) and worry as the second mediator (M2) in the mediation chain. Our results showed that unintentional MW was positively related to rumination (*β* = 0.34, *p* = 0.01, R^2^ = 0.12), and a high level of rumination predicted a high level of worry (*β* = 0.66, *p* < 0.0001, R^2^ = 0.42). Next, we computed the analyses for worry (M2) as the second mediator and found that worry was positively related to internalising symptoms (*β* = 0.50, *p* = 0.0006, R^2^ = 0.46).

**Fig. 1:**
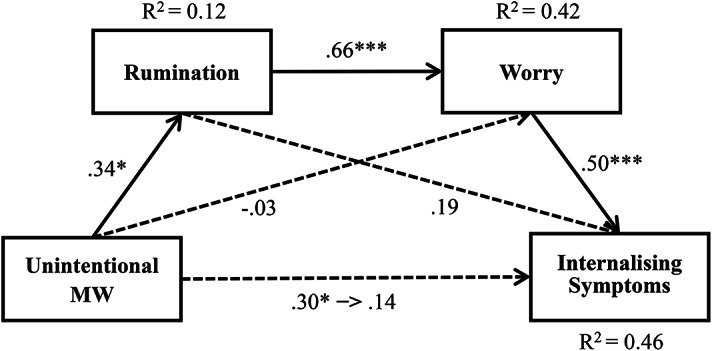
Mediation effect path of rumination and worry between unintentional MW and internalising symptoms. Note. Numbers are presented with standardised coefficients. *p < 0.05. **p < 0.01. ***p < 0.001.

Consistent with Hypothesis 1, unintentional MW is positively and indirectly related to internalising symptoms mediated by rumination and worry. The results of the mediation effect analysis showed that unintentional MW was not directly related to internalising symptoms (Effect = 0.0291, 95% CI [-0.0168, 0.0750]); however, rumination (M1) and worry (M2) mediated this relationship (Effect = 0.0235, 95% CI [0.0053, 0.0453]). Additionally, the indirect effect of unintentional MW on internalising symptoms only through rumination (Effect = 0.0134, 95% CI [-0.0019, 0.0375]) and worry (Effect = -0.0031, 95% CI [-0.0331, 0.0182]) was not significant. Hence, there was chain mediation of rumination and worry in the relationship between unintentional MW and internalising symptoms (see Fig. 1).

Finally, to confirm that the reverse order was not statistically supported, we examined the chain mediation of the model with worry as the first mediator (M1) and rumination as the second (M2) in the mediation chain. The indirect effect of unintentional MW on internalising symptoms mediated by worry (M1) and rumination (M2) (Effect = 0.0046, 95% CI [-0.0018, 0.0165]), only through worry (Effect = 0.0204, 95% CI [-0.0052, 0.0429]), and then, only through rumination (Effect = 0.0088, 95% CI [-0.0022, 0.0275]), was not significant in all cases.

### Chain mediation effects of rumination and worry on the relationship between unintentional future-oriented MW and internalising symptoms

By testing the mediation models, we first computed the model for rumination as the first mediator (M1) and worry as the second mediator (M2) in the mediation chain. Our results showed that unintentional future-oriented MW was positively related to rumination (*β* = 0.32, *p* = 0.0188, R^2^ = 0.10), and that a high level of rumination related to a high level of worry (*β* = 0.59, *p* < 0.0001, R^2^ = 0.45). Next, we computed the analyses for worry (M2) as the second mediator and found that worry was positively related to internalising symptoms (*β* = 0.47, *p* = 0.0014, R^2 ^= 0.45).

**Fig. 2:**
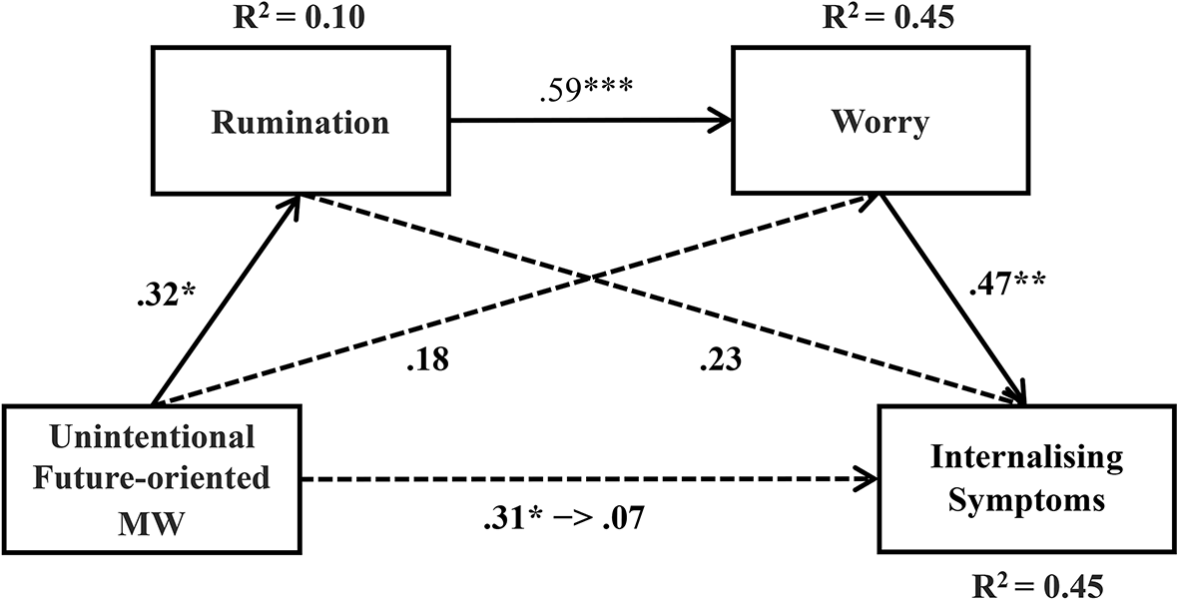
Mediation effect path of rumination and worry between unintentional future-oriented MW and internalising symptoms. Note. Numbers are presented with standardised coefficients. *p < 0.05. **p < 0.01. ***p < 0.001.

Consistent with Hypothesis 2, unintentional future-oriented MW was positively and indirectly related to internalising symptoms mediated by rumination and worry. The results of the mediation effect analysis showed that unintentional future-oriented MW was not directly related to internalising symptoms (Effect = 0.0192, 95% CI [-0.0435, 0.0819]), but rumination (M1) and worry (M2) mediated this relationship (Effect = 0.0245, 95% CI [0.0053, 0.0487]). Additionally, the indirect effect of unintentional future-oriented MW on internalising symptoms only through rumination (Effect = 0.0201, 95% CI [-0.0006, 0.0491]) and only through worry (Effect = 0.0233, 95% CI [-0.0005, 0.0475]) was all not significant. Hence, there was chain mediation of rumination and worry in the relationship between unintentional future-oriented MW and internalising symptoms (see Fig. 2).

Finally, to confirm that the reverse order was not statistically supported, we examined the serial mediation of the model, with worry as the first mediator (M1) and rumination as the second (M2) in the mediation chain. The indirect effect of unintentional future-oriented MW on internalising symptoms mediated by worry (M1) and rumination (M2) (Effect = 0.0142, 95% CI [-0.0002, 0.0427]), and then, only through rumination (Effect = 0.0059, 95% CI [-0.0097, 0.0241]) was all not significant.However, the indirect effect of unintentional future-oriented MW on internalising symptoms mediated only by worry (Effect = 0.0478, 95% CI [0.0145, 0.0815]) was significant.

### Chain mediation effects of rumination and worry on the relationship between unintentional vague MW and internalising symptoms

Furthermore, by testing the mediation models, we first computed the model for rumination as the first mediator (M1) and worry as the second mediator (M2) in the mediation chain. Our results showed that unintentionally vague MW was positively related to rumination (*β* = 0.38, *p* = 0.0041, R^2^ = 0.15), and a high level of rumination predicted a high level of worry (*β* = 0.65, *p* < 0.0001, R^2^ = 0.42). Next, we computed the analyses for worry (M2) as the second mediator and found that worry was positively related to internalising symptoms (*β* = 0.49, *p* = 0.0007, R^2^ = 0.45).

**Fig. 3:**
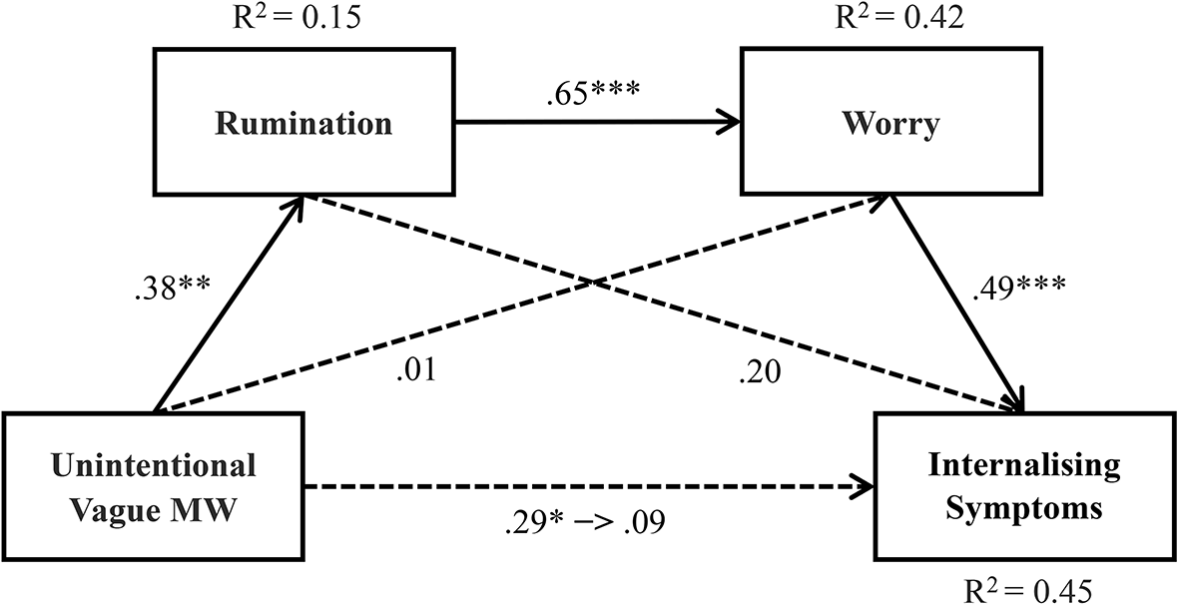
Mediation effect path of rumination and worry between unintentional vague MW and internalising symptoms. Note. Numbers are presented with standardised coefficients. *p < 0.05. **p < 0.01. ***p < 0.001.

Consistent with Hypothesis 3, unintentional vague MW is positively and indirectly related to internalising symptoms mediated by rumination and worry. The results of the mediation effect analysis showed that unintentional vague MW was not directly related to internalising symptoms (Effect = 0.0267, 95% CI [-0.0380, 0.0913]); however, rumination (M1) and worry (M2) mediated this relationship (Effect = 0.0346, 95% CI [0.0108, 0.0631]). Additionally, the indirect effect of unintentional vague MW on internalising symptoms only through rumination (Effect = 0.0223, 95% CI [-0.0006, 0.00604]) and worry (Effect = 0.0007, 95% CI [-0.0391, 0.0303]) was not significant. Hence, there was chain mediation of rumination and worry in the relationship between between unintentionally vague MW and internalising symptoms (see Fig. 3).

Finally, to confirm that the reverse order was not statistically supported, we examined the serial mediation of the model, with worry as the first mediator (M1) and rumination as the second (M2) in the mediation chain. The indirect effect of unintentionally vague MW on internalising symptoms mediated by worry (M1) and rumination (M2) (Effect = 0.0086, 95% CI [-0.0020, 0.0272]), worry (Effect = 0.0353, 95% CI [-0.0036, 0.0682]), and rumination (Effect = 0.0136, 95% CI [-0.0010, 0.0432]) was not significant in all cases.

### Chain mediation effects of rumination and worry on the relationship between unintentional negative MW and internalising symptoms

By testing the mediation models, we first computed the model for rumination as the first mediator (M1) and worry as the second mediator (M2) in the mediation chain. Our results showed that unintentional negative MW was positively related to rumination (*β* = 0.37, *p* = 0.0052, R^2^ = 0.14), and a high level of rumination predicted a high level of worry (*β* = 0.56, *p* < 0.0001, R^2^ = 0.46). Next, we computed the analyses for worry (M2) as the second mediator and found that worry was positively related to internalising symptoms (*β* = 0.47, *p* = 0.0017, R^2^ = 0.45).

**Fig. 4:**
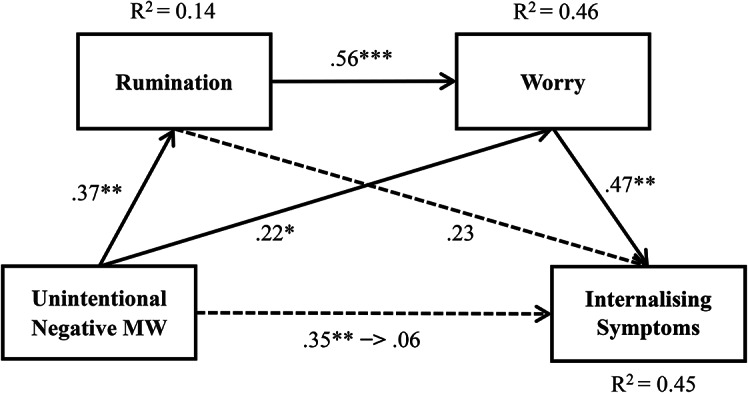
Mediation effect path of rumination and worry between unintentional negative MW and internalising symptoms. Note. Numbers are presented with standardised coefficients. *p < 0.05. **p < 0.01. ***p < 0.001.

Consistent with Hypotheses 4, unintentional negative MW was positively and indirectly related to internalising symptoms mediated by rumination and worry. The results of the mediation effect analysis showed that unintentional negative MW was not directly related to internalising symptoms (Effect = 0.0323, 95% CI [-0.0855, 0.1502]), but rumination (M1) and worry (M2) mediated this relationship (Effect = 0.0496, 95% CI [0.0129, 0.1152]). Further, an indirect effect of unintentional negative MW on internalising symptoms only through rumination (Effect = 0.0428, 95% CI [-0.0024, 0.1035]) was not significant, but only through worry (Effect = 0.0532, 95% CI [0.0034, 0.1217]) was significant. Hence, there was chain mediation of rumination and worry in the relationship between unintentional negative MW and internalising symptoms (see Fig. 4).

Finally, to confirm that the reverse order was not statistically supported, we examined the serial mediation of the model, with worry as the first mediator (M1) and rumination as the second (M2) in the mediation chain. The indirect effect of unintentional negative MW on internalising symptoms mediated by worry (M1) and rumination (M2) (Effect = 0.0299, 95% CI [-0.0006, 0.0816]), and then, only through rumination (Effect = 0.0128, 95% CI [-0.0193, 0.0458]) was not significant. However, the indirect effect of unintentional negative MW on internalising symptoms mediated only by worry (Effect = 0.01027, 95% CI [0.0388, 0.1952]) was significant.

## Discussion

The current study examined the chain mediating roles of rumination and worrying in the relationship between dysfunctional MW and internalising symptoms. The results indicated that rumination and worry mediate the relationship between unintentional MW, encompassing vague, future-oriented, negative content, and internalising symptoms. In addition, a pathway involving worry alone was also identified in the association between unintentional negative MW and internalising symptoms.

The combination of quantitative and qualitative characteristics of individuals’ MW trait is associated with internalizing symptoms through maladaptive emotion regulation strategies. This funding supported the framework used in previous studies and emphasised that MW, rumination, and worry should be accessed as different constructs^2,30^. MW was more negative, past-oriented, and self-related in patients with MDD than in controls^48^. Our funding suggests that, rather than directly related to internalising symptoms, high-frequency dysfunctional MW may indirectly relate to internalising symptoms positively through maladaptive emotion regulation strategies of rumination and worry. Thus, individuals’ MW tendencies across the trait continuum, categorized by their quantity (frequency) and quality (intentionality and content), are involved in emotion dysregulation, contributing to internalizing symptoms. Future research should employ longitudinal studies to explore the temporal dynamics of the role of emotion dysregulation in MW and internalizing symptoms.

Rumination and worry may play different roles in the relationship between dysfunctional MWs and internalizing symptoms. Rumination is defined as thoughts that direct attention to self-relevant information, motivated by threats or loss^37^. It is possible that dysfunctional MW is more likely to direct attention to self-relevant information motivated by loss and threat to the self as opposed to unintentionally generated internally oriented thoughts. In contrast, worry could result from a cognitive response to perceived negative self-referential information and the avoidance of threats. The worry avoidance theory emphasises that worry is a cognitive activity that diverts attention from more emotionally distressing content as opposed to imaginative and concrete information processing^39^. Furthermore, worry is more strongly associated with internalising symptoms, including depression and anxiety, than rumination^40^. In summary, rumination and worry may play different roles in the association between dysfunctional MWs and internalising symptoms.

Remarkably, the independent role of worry mediates the association between unintentional negative MW and internalizing symptoms, functioning as a maladaptive emotion regulation strategy by predicting threats and avoiding negative content. Our findings suggest that negative unintentional MW is linked to internalizing symptoms through both rumination and worry pathways, as well as worry-only pathways. The worry avoidance theory emphasizes that worry diverts attention from emotionally distressing content, as opposed to imaginative or concrete information processing^39^. According to this theory, worry centers on the perception of threats, making it a response-focused strategy aimed at avoiding emotional distress. Based on our findings, unintentional negative MW may independently predict threats and strengthen the avoidance of negative content. Future research should manipulate MW content to examine its effects on worry and rumination.

The present study has several limitations that warrant consideration and point to directions for future research. First, its cross-sectional design assumes theoretical sequential relationships between MW, trait rumination, trait worry, and the internalizing symptoms of anxiety and depression. To clarify these relationships, longitudinal designs, such as experience sampling in daily life, are needed to examine temporal dynamics, causal pathways, and changes over time. Second, the sample consisted primarily of undergraduate and graduate students, limiting the generalizability of the findings. Future research should include more diverse populations and clinical samples, such as individuals with anxiety, depression, or other mental disorders, to better understand how these processes manifest across different contexts and to inform targeted interventions aimed at reducing dysfunctional MW. Third, while participants self-reported no history of mental illness, we did not collect clinical diagnostic information. Incorporating clinical evaluations in future studies could address this issue. Furthermore, the reliance on self-reported measures for MW poses challenges, as such measures are susceptible to response biases. Employing neurophysiological, or physiological methods—such as EEG, fMRI, pupillometry, or heart rate variability—could provide objective insights into the neurophysiological mechanisms underlying MW and its relationship with emotional regulation processes like rumination and worry. Finally, this study examined trait worry and rumination without accounting for state-level measures, leaving room for future investigations to explore the dynamic interplay between state-level worry, rumination, and MW. Addressing these limitations through methodological advancements and diverse study designs will enhance our understanding of MW and its implications for mental health.

## Conclusion

This study explored the mediating roles of rumination and worry in the relationship between dysfunctional MW and internalizing symptoms. Our findings suggested that both rumination and worry mediate the relationship between unintentional MW, particularly characterized by vague, future-oriented, negative content, and internalizing symptoms. Notably, worry alone also mediated this relationship when unintentional negative MW was involved, underscoring its independent role in linking the frequency of such MW to internalizing symptoms. The combination of quantitative and qualitative MW characteristics contributes to internalizing symptoms through the maladaptive emotion regulation strategies of rumination and worry. Future research should manipulate the intentionality and content dimensions of MW to examine the mediating roles of rumination and worry in the relationship between MW and internalizing symptoms, with a particular focus on their temporal dynamics.

## Data Availability

The dataset analyzed during the current study is available from the corresponding author upon reasonable request.
